# A UPLC-MS/MS Method for Simultaneous Determination of Free and Total Forms of a Phenolic Acid and Two Flavonoids in Rat Plasma and Its Application to Comparative Pharmacokinetic Studies of *Polygonum capitatum* Extract in Rats

**DOI:** 10.3390/molecules22030353

**Published:** 2017-02-25

**Authors:** Yong Huang, Hui-Yuan Sun, Xiao-Li Qin, Yong-Jun Li, Shang-Gao Liao, Zi-Peng Gong, Yuan Lu, Yong-Lin Wang, Ai-Min Wang, Yan-Yu Lan, Lin Zheng

**Affiliations:** 1 Provincial Key Laboratory of Pharmaceutics in Guizhou Province, Guizhou Medical University, 4 Beijing Road, Guiyang 550004, China; mailofhy@126.com (Y.H.); lshangg@163.com (S.-G.L.); gzp4012607@126.com (Z.-P.G.); 18798090340@163.com (Y.L.); ylwang_gmc@163.com (Y.-L.W.); 2 School of Pharmacy, Guizhou Medical University, 4 Beijing Road, Guiyang 550004, China; sunhuiyuanshy@163.com (H.-Y.S.); qinxiaoli2017@163.com (X.-L.Q.); liyongjun026@126.com (Y.-J.L.); gywam100@163.com (A.-M.W.); 3 National Engineering Research Center of Miao’s Medicines, 4 Beijing Road, Guiyang 550004, China; 4 Engineering Research Center for the Development and Application of Ethnic Medicine and TCM, Ministry of Education, Guizhou Medical University, 4 Beijing Road, Guiyang 550004, China

**Keywords:** *Polygonum capitatum* extract, phenolic acids, flavonoids, UPLC-ESI-MS/MS, pharmacokinetics, hydrolysis

## Abstract

The principal active constituents of *Polygonum capitatum* are phenolic acids and flavonoids, such as gallic acid, quercitrin, and quercetin. The aim of this study was to develop and validate a method to determine the three constituents and the corresponding conjugated metabolites of *Polygonum capitatum* in vivo and to conduct pharmacokinetic studies on the herb, a well-known Miao medicinal plant in China. Gallic acid, quercitrin, and quercetin were analysed by ultra-performance liquid chromatography-electrospray ionization-tandem mass spectrometry (UPLC-ESI-MS/MS). Protein precipitation in plasma samples was performed using methanol. For the determination of total forms of analytes, an additional process of hydrolysis was conducted using β-glucuronidase and sulphatase. The analytes were separated on a BEH C_18_ column (50 mm × 2.1 mm; i.d., 1.7 μm) and quantified by multiple reaction monitoring (MRM) mode. The linear regression showed high linearity over a 729-fold dynamic range for the three analytes. The relative standard deviations of intra- and inter-day measurements were less than 9.5%, and the method was accurate to within −11.1% to 12.5%. The extraction recoveries for gallic acid, quercitrin, and quercetin were 94.3%–98.8%, 88.9%–98.8%, and 95.7%–98.5%, respectively. All samples were stable under short- and long-term storage conditions. The validated method was successfully applied to a comparative pharmacokinetic study of gallic acid, quercitrin, and quercetin in their free and total forms in rat plasma. The study revealed significantly higher exposure of the constituents in total forms for gallic acid and quercetin, while quercitrin was detected mainly in its corresponding free form in vivo. The established method was rapid and sensitive for the simultaneous quantification of free and total forms of multiple constituents of *Polygonum capitatum* extract in plasma.

## 1. Introduction

**Figure 1:**
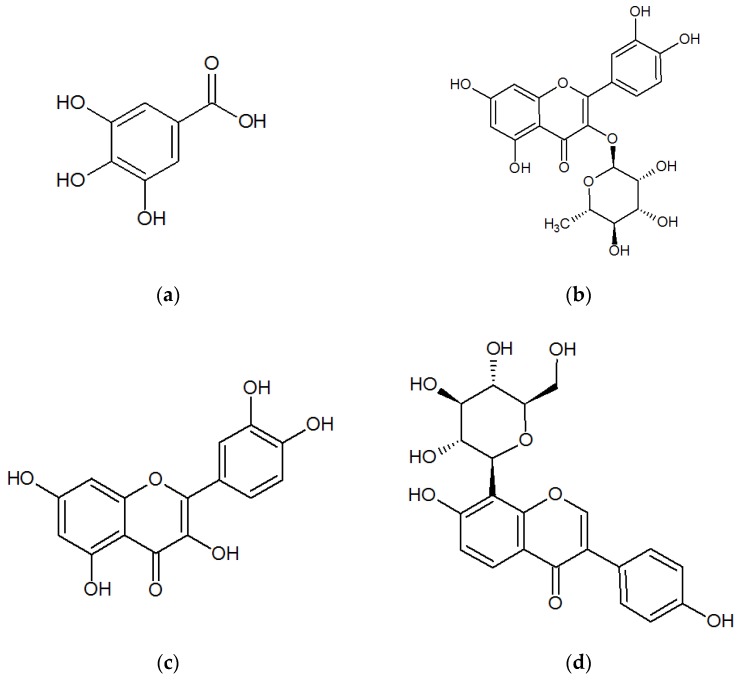
Chemical structures of (**a**) gallic acid; (**b**) quercitrin; (**c**) quercetin; and (**d**) puerarin (I.S.).

Traditional Chinese medicine (TCM) has been used for a long time in clinical practice in China, especially for treating complex and chronic disorders. Further, there is a long history of the use of plant-based remedies in a variety of TCM and ethnic medicines for the treatment of urinary tract infections (UTIs). Among these plants, *Polygonum capitatum*, a well-known Miao’s medicinal plant, has been widely used because of its considerable therapeutic efficacy for the treatment of various urologic disorders, including urinary calculus and UTIs by the Miao people in Southwest China [1,2]. Several *P. capitatum*-based drugs (e.g., Relinqing^®^ Granule and Milin^®^ Capsule) have been approved by China Food and Drug Administration, and Relinqing^®^ Granule has been officially listed in the Chinese Pharmacopoeia since 2010 [3]. Some pharmacological studies have shown that aqueous or ethanolic extracts of *P. capitatum* possess antibacterial, analgesic, anti-inflammatory, hypothermic, diuretic, and antioxidative activities [4,5,6,7], whereas some published investigations have suggested that the clinical therapeutic efficacy of *P. capitatum* should be attributed to the presence of constituent flavonoids, gallic acid, and its analogues, lignin, triterpenoids, and fatty acid esters [8,9,10]. Phenolic acids and flavonoids [11] are thought to contribute majorly to the clinical therapeutic efficacy of *P. capitatum* [8,12,13], and some recent studies have shown gallic acid, quercitrin, and quercetin (Chemical structures shown in Figure 1) to be the top three constituents of phenolic acids and flavonoids in the crude *P. capitatum* herb and its prescriptions [14,15].

Previous studies have shown that phenolic acids and flavonoids are widely transformed to their corresponding glucuronide and/or sulphate conjugates (phase II metabolites) during absorption and/or circulation after oral administration of *P. capitatum* extract [16,17,18,19,20]. The conjugation process can be attributed wholly or partially to the action of the bioactive constituents in vivo. In addition to the attention focused on drug-drug interactions in therapy, drug/metabolite(s) interactions should be noted in pharmacokinetic mass balance studies, especially for cases in which the regeneration of a constituent from the conjugated metabolites is significant [19]. Therefore, it is important to characterise and compare the pharmacokinetics and metabolic fates of free forms and their conjugates in vivo, especially in cases of complex and multicomponent traditional Chinese medicines.

Gallic acid, quercitrin, and quercetin are the three major representative constituents of phenolic acids, flavonoid glycosides, and aglycones with pharmacological activities in *P. capitatum*. Although many studies on the quantitative and qualitative analyses of these compounds have been published [13,14,15,21,22], there are few published studies providing integrated data on their pharmacokinetic properties, and on the simultaneous determination of their free and total forms, which may contribute to the elucidation of *P. capitatum* effects in vivo. In order to determine their total concentrations, the conjugated metabolites can be hydrolysed to their respective free forms by treatment with β-glucuronidase and sulphatase, which are mild and specific hydrolysis enzymes [19,23]. Analytical methods for individual determination of gallic acid, quercitrin, and quercetin in biological samples, including high-performance liquid chromatography (HPLC) with MS detection, have been described in several studies [24,25,26]. However, currently, there is no established method for the simultaneous determination of the three bioactive free constituents of *P. capitatum* or their conjugated metabolites in plasma for a comparative pharmacokinetic study.

Therefore, the objective of this study was to develop and validate a method for the simultaneous measurement of plasma levels of gallic acid, quercitrin, and quercetin and their conjugated metabolites by using rapid and sensitive ultra-performance liquid chromatography-electrospray ionization-tandem mass spectrometry (UPLC-ESI-MS/MS). This method was applied to determine the pharmacokinetics of these constituents after oral administration of *P. capitatum* extract in rats.

## 2. Results and Discussion

### 2.1. Chromatography and Mass Spectrometry

Analytes with relatively low concentrations are usually present in a complex matrix environment, especially in biological samples. In this study, we developed a sensitive, specific, and reliable UPLC-ESI-MS/MS system to quantify the plasma levels of gallic acid, quercetin, and puerarin in *P. capitatum* extract in a single assay following oral administration in rats. Addition of 0.1% formic acid to the mobile phase led to a satisfactory peak symmetry and good resolution, and significantly enhanced sensitivity and reduced the analysis time with a 50-mm-long column.

The precursor and product ions of these analytes and puerarin (internal standard (I.S.)) were optimized by using the standard solution in the multiple reaction monitoring (MRM) mode. The three analytes and I.S. showed different response values in the positive and negative ion modes. Ionization of quercetin, puerarin, and I.S. yielded much stronger relative intensities in the positive ion mode, while the ionization of gallic acid showed much stronger relative intensities in the negative ion mode. In order to achieve the low detection limits required, a method involving both positive and negative ionization modes was selected to allow for simultaneous detection.

### 2.2. Sample Preparation and I.S. Selection

Several sample-processing methods were tested, and the recovery was found to be poor with ethyl acetate extraction and protein precipitation with acetonitrile or perchloric acid. Although protein removal from the plasma samples could be performed effectively using solid-phase extraction (SFE) columns, the procedure proved to be complicated, expensive, and time-consuming. A simple one-step plasma protein-precipitating procedure with methanol afforded acceptable recovery for the analytes and I.S., with clear sample solutions and minor interfering peaks. The strong polarity made it difficult to rapidly free the phenolic acid and flavonoids from the plasma protein; therefore, several acid solvents were tested to improve the extraction efficiency, including acetic acid (1%, *v*/*v*), formic acid (1%, *v*/*v*), and hydrochloric acid (0.1, 0.2, 0.4 mol/L). Consequently, the addition of 50 µL of formic acid (1%, *v*/*v*) to 100 µL of rat plasma was found to result in high analyte recovery.

Selection of a suitable I.S. is a key factor in biological sample analysis. A stable isotope-labelled analyte is considered ideal, but it is difficult to obtain such compounds. A feasible alternative is a compound with similar physicochemical properties to the analyte. During the method development phase in our study, various chemicals such as icariin, kaempferol, hyperin, naringenin, and puerarin were separately tested. However, most of them were unsuitable because of endogenous interference or retention time. Puerarin was chosen as the I.S. because it separates well from the analytes and does not exist endogenously in the plasma. Moreover, the elution time of puerarin was shorter than that of the last analyte, thereby allowing clean chromatography.

### 2.3. Method Validation

#### 2.3.1. Selectivity, Matrix Effect, and Recovery

**Figure 2:**
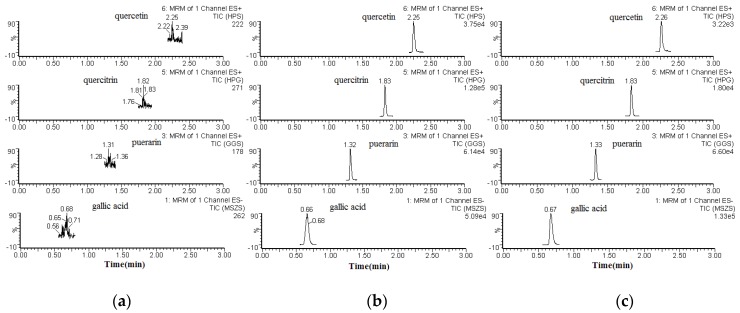
Chromatograms of the analytes and puerarin (I.S.) in rat plasma. Blank plasma sample (**a**); a plasma sample spiked with gallic acid, quercitrin, and quercetin, or puerarin (I.S.), respectively (**b**); and a plasma sample obtained from a rat 60 min after oral administration of *P. capitatum* extract (**c**).

The specificity of the method was determined by comparing the chromatograms of blank (unspiked) plasma with that of the corresponding spiked plasma. Typical chromatograms of blank plasma (a), spiked plasma (b), and plasma from a pharmacokinetic study (c) are shown in Figure 2. The retention times for gallic acid, quercitrin, quercetin, and puerarin (I.S.) were 0.66, 1.83, 2.25, and 1.32 min, respectively. None of the analytes showed any endogenous interference under the UPLC-ESI-MS/MS conditions.

Regarding the matrix effects, all ratios (A/B × 100)% were between 90.9% and 100.5%, indicating that no significant co-elution, i.e., endogenous substances, interfered with the ionization of the analytes. The matrix effect of puerarin was 96.8%. The extraction recoveries ranged from 88.9% to 98.8%. All of these values were within acceptable ranges (Table 1).

#### 2.3.2. Linearity and Lower Limit of Quantification (LLOQ)

Typical equations for calibration curves and LLOQs of the three analytes are shown in Table 2. The precision and accuracy at the LLOQ of the three were acceptable with an RSD of 15% and an RE within ±15%. The lower limits of quantification (LLOQ) for gallic acid, quercitrin, and quercetin were 0.178 µg/mL, 0.007 µg/mL, and 0.037 µg/mL, respectively.

#### 2.3.3. Precision and Accuracy

As shown in Table 3, the results for intra- and inter-day precision and accuracy for the three analytes indicated that the intra- and inter-day RSDs were all less than 9.5% and 8.2%, respectively, while the corresponding REs ranged from −11.1% to 12.5%. These data suggest that both precision and accuracy achieved with this method are acceptable.

#### 2.3.4. Stability

The three analytes were stable under all testing conditions, including short-term storage, long-term storage, freeze-thaw cycling, and post-preparative storage (Table 4). The REs calculated from the QC experiments under all testing conditions ranged from −7.8% to 13.1%.

### 2.4. Pharmacokinetics Study

**Figure 3:**
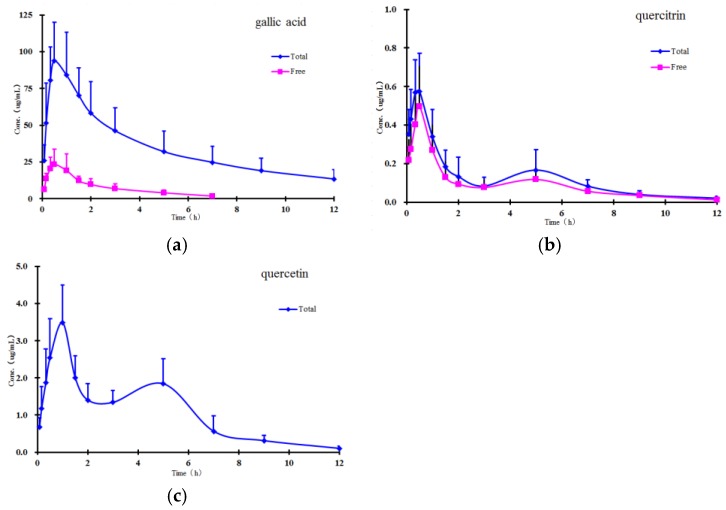
Mean plasma concentration-time curves of (**a**) gallic acid; (**b**) quercitrin; and (**c**) quercetin after oral administration of *P. capitatum* extract (equivalent to 21.35 mg/kg of gallic acid, 2.17 mg/kg of quercitrin, and 0.39 mg/kg of quercetin, respectively) to rats (*n* = 6).

The method validated in the present study was successfully applied to study the pharmacokinetics of free and total forms of gallic acid, quercitrin, and quercetin in rat plasma after oral administration of *P. capitatum* extract. The mean plasma concentration-time profiles of free and total forms are presented in Figure 3, and the results show that the free constituents and total conjugates (except conjugate quercetin) can be detected immediately in plasma within 10 minutes after oral administration, indicating that the precursors could be quickly absorbed and/or metabolized in the gastrointestinal tract. In comparison with the amounts of their corresponding glucuronide and/or sulphate conjugates in plasma, the proportions of free forms were relatively small, and the free form of quercetin was undetectable in plasma samples at all the sampling time points in the established analytical method.

Double absorption maxima for the quercetin conjugate are distinctly observed in the concentration-time profile, and the result might be presumably due to enterohepatic circulation, transformation of different ingredients, double-site absorption, and intestinal efflux [27]. The conjugates are first transported from the liver to the small intestine via the bile duct, where they are largely subsequently reabsorbed through the cavity of the gastrointestinal tract into the portal blood circulation [28]. Secondly, in addition to the conjugated forms, flavonoids also undergo phase I hydrolysis metabolism; for example, quercitrin transformed to quercetin, and then quercetin could also undergo glucuronidation. Therefore, mutual transformation could be one of the factors responsible for the double absorption maxima phenomenon [29]. Additionally, the drugs may show double-site absorption. For example, when the *P. capitatum* extract was administered directly into the stomach, the first peak represented the absorption of the ingredients in the stomach. The stomach is totally emptied within 4–6 h, and the ingredients continue down to the intestine in the alimentary canal; Figure 3 shows that the second peaks for total quercetin appeared at about 5 h, which was probably caused by intestinal absorption. Finally, some intravenously administered drugs can pass through the gastrointestinal membrane into the gastrointestinal tract. Since flavonoids have numerous efflux pumps, efflux could also be responsible for reabsorption of the drug and the double maxima.

The main non-compartmental pharmacokinetic parameters are summarized in Table 5. From the table, the parameters showing notable increments (AUC and C_max_) showed higher exposure of gallic acid conjugate than its corresponding precursor, which demonstrated that phenolic acids in *P. capitatum* extract may be commonly present as conjugates in vivo after administration. Meanwhile other significantly altered parameters (e.g., MRT) indicate the changes in the residence characteristics of phenolic acids and flavonoids owing to their rapid and extensive conjugating biotransformations. However, this had a relatively small effect on the AUC and C_max_ differences of quercitrin between the free and total forms (ratio, about 1:1.3 and 1:1.2), indicating likely minimal biotransformation in the form of flavonoid glycoside.

Quercitrin and quercetin showed another unexpected pharmacokinetic behaviour in this study: the pharmacokinetic parameters of quercetin showed much higher exposure than quercitrin did in their respective total forms, although the latter was present in much larger amounts than the former (approximately 5.5-fold by contents) in *P. capitatum* extract. According to the related reports [20,30,31], this result could imply two inferences: (a) quercitrin can hardly be absorbed as a flavonoid glycoside in the gastrointestinal tract; (b) the hydrolysed metabolite of quercitrin—quercetin—is most likely to be the supplementary to increase the exposure of quercetin in vivo. Therefore, it was suggested that mutual biotransformations of constituents seem likely to occur before and after the absorption when *P. capitatum* extract was orally administered.

## 3. Materials and Methods

### 3.1. Materials and Reagents

Gallic acid, quercetin, and puerarin were obtained from the National Institutes for Food and Drug Control (Beijing, China). The structures of gallic acid, quercetin, and puerarin (internal standard [I.S.]) are shown in Figure 1. The purity of these reference standards was more than 98.0%. β-Glucuronidase (type HP-2, from *Helix pomatia*) and sulphatase (type H-1 from *Helix pomatia*) were supplied by Sigma (St. Louis, MO, USA). HPLC-grade acetonitrile was purchased from Merck Co. (Darmstadt, Germany). HPLC-grade formic acid was supplied by Dikma (Richmond Hill, NY, USA). Distilled water was obtained from Watsons Group Co. Ltd. (Hong Kong, China). All other chemicals were of analytical grade.

Whole *P. capitatum* plants were collected from the Good Agricultural Practice (GAP) base of Touhualiao in Shibing (Guizhou, China), and a voucher specimen (accession number, PC20101201) was deposited at the herbarium of Guizhou Medical University, Guiyang, China. The plant material was sun-dried and ground.

*P. capitatum* extract was prepared according to the Chinese pharmacopeia [3] as follows: 1250 g of dried medicinal parts of *P. capitatum* was accurately weighed and extracted twice with water (5 L of water each time). Aqueous extracts were combined and then the supernatant was evaporated to dryness (182 g) for administration. Gallic acid, quercitrin, and quercetin contents in the extract were determined according to a previously described method [15]. Briefly, the chromatographic and mass spectrometric conditions were optimized to achieve an excellent chromatographic resolution and a satisfactory MS response on the ACQUITY^TM^ UPLC system (Waters Corp., Milford, MA, USA).

The contents of gallic acid, quercitrin, and quercetin in the extract were 3.05%, 0.31%, and 0.056%, respectively.

### 3.2. Apparatus and Operation Conditions

#### 3.2.1. Ultra-Performance Liquid Chromatography

An ACQUITY™ UPLC system with an auto-sampler was used. The analytical column used was an ACQUITY™ UPLC BEH C_18_ column (50 mm × 2.1 mm, i.d., 1.7 µm; Waters Corp., Milford, MA, USA) to which a Waters Van Guard BEH C_18_ pre-column (5 mm × 2.1 mm, i.d., 1.7 µm; Waters Corp.) filter unit was added. Analysis was carried out with an elution gradient of (A) acetonitrile and (B) water (both containing 0.1% formic acid) at a flow rate of 0.35 mL/min as follows: 0–1.5 min (5%–30% A), 1.5–3.0 min (30%–90% A), and 3.0–3.5 min (90%–5% A). The column and auto-sampler were maintained at 45 °C and 15 °C, respectively. The injection volume was 2 µL.

#### 3.2.2. Mass Spectrometry

**Figure 4:**
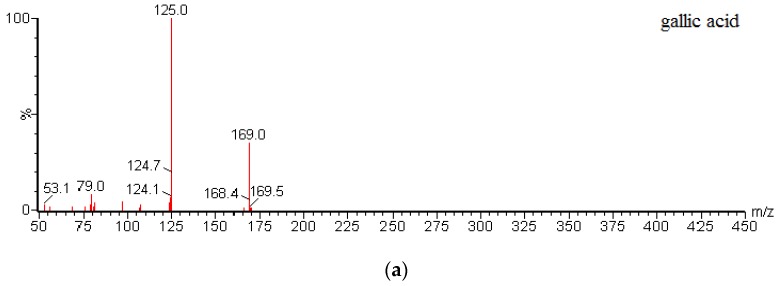

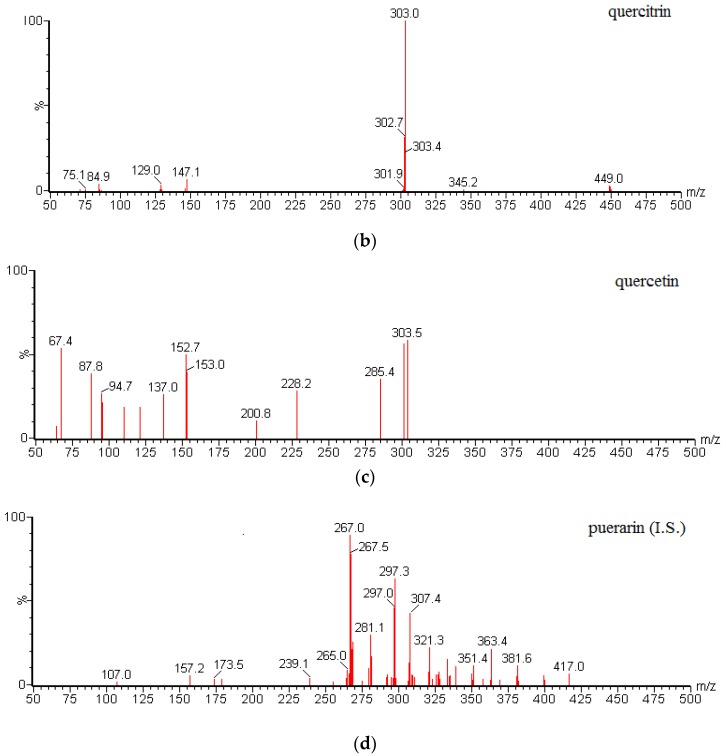
MS/MS spectra of (**a**) gallic acid; (**b**) quercitrin; (**c**) quercetin; and (**d**) I.S.

Mass spectrometric detection was performed using a Waters ACQUITY™ TQD triple quadrupole tandem mass spectrometer (Waters Corp., Manchester, UK) with an ESI interface in the positive/negative ionization mode. The MS conditions were as follows: capillary voltage, 3.0 kV; desolvation gas flow, 650 L/h nitrogen; and cone gas flow, 50 L/h nitrogen. The collision gas (Ar) for MS/MS was maintained at 0.16 mL/min for collision-induced dissociation (CID). The source and desolvation gas temperatures were 120 °C and 350 °C, respectively. The MRM mode was applied for quantification. The optimal MRM parameters of the analytes and I.S. are given in Table 6. All data were acquired using Masslynx™ V4.1 software and processed using Quanlynx™ V4.1 (Waters Corp., Milford, MA, USA). MS/MS spectra of gallic acid, quercetin, and puerarin (I.S.) are shown in Figure 4.

### 3.3. Stock Solutions, Standards, and Quality Control Samples

Stock solutions of gallic acid, quercitrin, and quercetin were prepared by dissolving the proper amounts of each standard substance in 10 mL of methanol to yield concentrations of 0.68, 1.02, and 1.01 mg/mL, respectively. Stocks were serially diluted with the initial mobile phase to obtain working solutions from which standard curves for each flavonoid were generated. Mixtures containing each of the three standards were also prepared and serially diluted to provide working solutions used for validation (quality control (QC)) experiments. The I.S. working solution (20 µg/mL) was prepared by dissolving the proper amount of puerarin in methanol. All of the stocks and working solutions were stored at 4 °C and brought to room temperature before use.

### 3.4. Sample Preparation

#### 3.4.1. Determination of Free Forms in Rat Plasma

Ten microliters of the I.S. solution (puerarin, 20 µg/mL in methanol) and 50 µL of 1% (*v*/*v*) formic acid were added to 100 µL of rat plasma, and vortexed for 1 min. Proteins were precipitated after addition of 400 µL of methanol and sonication for 5 min. The mixture was then centrifuged at 15,000× *g* for 5 min at 4 °C. The upper organic phase was transferred into tubes and evaporated to dryness under a gentle stream of nitrogen at 48 °C. The residues were redissolved in 200 µL of the initial mobile phase before UPLC-ESI-MS/MS analysis.

#### 3.4.2. Determination of Total Forms in Rat Plasma

Sample plasma (100 µL) was incubated with 10 µL of enzyme (β-glucuronidase 8000 units/mL and sulphatase 2000 units/mL in 4% vitamin C solution) at 37 °C for 1 h [27]. The processing thereafter was performed as described above.

### 3.5. Method Validation

The method to quantify analyte concentrations in rat plasma was fully validated for selectivity and matrix effect, linearity, accuracy, precision, recovery, and stability according to the US Food and Drug Administration Bio-analytical Method Validation Guide [32].

#### 3.5.1. Selectivity, Matrix Effect, and Recovery

To investigate selectivity, chromatograms of plasma samples obtained from untreated rats were compared to those of the plasma samples obtained after oral administration of the extract. Matrix effects were determined by comparing the peak areas of analytes in the samples spiked after extraction (A) with those of analyte standard solutions dried directly and reconstituted with the same volume of the initial mobile phase (B). Three concentrations of the analytes, each in five replicates, were studied. When the ratios (A/B × 100)% of the analytes and the I.S. solution were between 85% and 115%, the matrix effect was considered negligible. The same method was also applied for the I.S.

The extraction recoveries of gallic acid, quercitrin, and quercetin at three QC levels were determined by comparing the peak areas of analytes obtained from plasma samples with the analytes spiked before extraction with those of the analytes spiked after extraction, which represented 100% recovery. The extraction recovery of the I.S. was determined similarly.

#### 3.5.2. Linearity and Lower Limit of Quantification

Linearity in rat plasma was evaluated on three consecutive days by using the following calibration standards: (1) gallic acid: 0.178, 0.533, 1.599, 4.798, 14.393, 43.179, and 129.537 µg/mL; (2) quercitrin: 0.007, 0.020, 0.059, 0.177, 0.532, 1.596, and 4.789 µg/mL; and (3) quercetin: 0.037, 0.112, 0.335, 1.004, 3.011, 9.032, and 27.096 µg/mL. Calibration curves for each analyte were generated by plotting the ratio of the peak area of the constituent to puerarin (*y*) versus the nominal concentration (*x*) of the standard by using 1/*x*-weighted least-square linear regression. The concentrations of the constituents in QC or unknown samples were calculated by linear interpolation from the calibration curves.

The lower limit of quantification (LLOQ) was defined as the lowest concentration on the calibration curve with accuracy (relative error [RE]; see equation below) within ±20% and precision (relative standard deviation (RSD)) less than 20%.

#### 3.5.3. Precision and Accuracy

Precision and accuracy were assessed on three consecutive days by using mixtures containing low, medium, or high concentrations of the QC samples (gallic acid: 0.533, 4.798 or 43.179 µg/mL; quercitrin: 0.02, 0.177 or 1.596 µg/mL; quercetin: 0.112, 1.004 or 9.032 µg/mL). Precision was expressed as the RSD, and accuracy was calculated using the following equation: ((mean measured concentration − spiked concentration)/(spiked concentration)) × 100%. Intra-day and inter-day precision and accuracy were determined by repeated analysis of the QC samples.

#### 3.5.4. Stability

The stability of the analytes in rat plasma was evaluated using mixtures containing low, medium, or high concentrations of the QC samples (*n* = 5 for each concentration level). Sample stability was tested under the following conditions: (1) room temperature for 6 h (short-term); (2) storage at −20 °C for 14 days (long-term); (3) three freeze-thaw cycles (freeze-thaw); and (4) storage in the auto-sampler at 15 °C for 8 h (post-preparative storage).

### 3.6. Pharmacokinetic Study

Twelve Sprague-Dawley rats (six males, six females, weighing 220–250 g) were obtained from the Experiment Animal Center (Guizhou Medical University, Guizhou, China). All animal maintenance and experimental studies were based on the guidelines of the National Institutes of Health for the Care and Use of Animals, and were approved by the Experiment Animal Center of Guizhou Medical University. All rats were kept in a breeding room with a controlled environment for one week before the experiment, and fed standard lab food and water ad libitum. After overnight fasting, the rats were divided into two groups (three males and three females in each group) and given *P. capitatum* extract at a dose of 700 mg/kg (i.g.) (equivalent to 21.35 mg/kg of gallic acid; 2.17 mg/kg of quercitrin; and 0.392 mg/kg of quercetin, respectively). Then the rats were fasted again for the first 2 h after administration with free access to water. Blood samples (approximately 0.2 mL) were collected via the caudal vein into centrifuge tubes coated with heparin before extract administration and at 0.083, 0.167, 0.333, 0.5, 1, 2, 3, 5, 7, 9, and 12 h after dosing. The samples were immediately centrifuged at 3000× *g* for 5 min, and the plasma was removed and frozen at −20 °C until analysis.

### 3.7. Statistical Analysis

Data are represented as mean ± standard deviation. To determine the pharmacokinetic parameters, the concentration-time data were analysed by DAS 2.1 Software (Mathematical Pharmacology Professional Committee of China, Shanghai, China). Statistical analysis was performed using the statistical software package Statistical Product and Service Solutions (SPSS 11.5, SPSS Inc., Chicago, IL, USA).

## 4. Conclusions

In this study, we developed and validated a simple, rapid, and sensitive UPLC-ESI-MS/MS method for the simultaneous determination of gallic acid, quercitrin, and quercetin and their conjugates in rat plasma following oral administration. This method was successfully applied to a comparative pharmacokinetic study of representative constituents from *P. capitatum* extract, and the results revealed pharmacokinetic profiles of the free and total forms of these analytes. The pharmacokinetic results indicated that the main bioactive constituents can be widely metabolized before and after absorption; therefore, the total forms other than the free ones might provide novel insight into the action mechanism of *P. capitatum*. Moreover, the established method and obtained results might also contribute to the assessment of safety and efficacy of *P. capitatum* in clinical practice.

## Figures and Tables

**Table 1:** Matrix effects and extraction recoveries of the analytes (*n* = 5).

Analytes	Spiked Conc. (µg/mL)	Matrix Effects (%)	RSD (%)	Recoveries (%)	RSD (%)
Gallic acid	0.533	94.2 ± 0.3	0.3	97.1 ± 1.2	1.2
	4.798	98.4 ± 1.6	1.6	94.3 ± 0.7	0.7
	43.179	96.3 ± 0.9	0.9	98.8 ± 0.2	0.2
Quercitrin	0.020	91.0 ± 3.4	3.7	88.9 ± 4.1	4.6
	0.177	93.7 ± 4.4	4.7	98.8 ± 2.0	2.0
	1.596	92.5 ± 1.9	2.1	95.1 ± 2.3	2.4
Quercetin	0.112	90.9 ± 4.7	5.2	95.7 ± 1.7	1.8
	1.004	95.2 ± 4.1	4.3	97.6 ± 2.4	2.5
	9.032	100.5 ± 3.0	3.0	98.5 ± 0.6	0.6

**Table 2:** Typical equations for calibration curves and lower limit of quantitation (LLOQ) (*n* = 3).

Analytes	Calibration Curves	Linear Range (µg/mL)	*r*^2^	LLOQ (µg/mL)
Gallic acid	*y* = 0.1314*x* + 0.1893	0.178–129.537	0.998	0.178
Quercitrin	*y* = 0.9007*x* + 0.0846	0.007–4.789	0.999	0.007
Quercetin	*y* = 0.0622*x* + 0.0576	0.037–27.096	0.998	0.037

**Table 3:** Summary of precision and accuracy of quality control samples added to rat plasma (*n* = 5).

Analytes	Spiked Conc. (µg/mL)	Intra-Day Precision (%, RSD)	Intra-Day Accuracy (%, RE)	Inter-Day Precision (%, RSD)	Inter-Day Accuracy (%, RE)
Gallic acid	0.533	1.9	7.4	1.2	12.5
	4.798	1.2	−6.5	0.7	−5.8
	43.179	1.2	3.4	0.8	2.0
Quercitrin	0.020	9.5	−3.2	8.2	−11.1
	0.177	4.5	1.3	0.9	1.8
	1.596	1.4	1.2	2.0	−3.5
Quercetin	0.112	1.5	−2.8	4.8	10.6
	1.004	1.6	−3.3	0.2	−5.7
	9.032	0.1	1.5	0.5	1.9

**Table 4:** Summary of stability of quality control samples added to rat plasma (*n* = 5).

Analytes	Spiked Conc. (µg/mL)	Stability (%, RE)			
		Short-Term	Long-Term	Freeze-Thaw	Post-Preparative
Gallic acid	0.533	−7.1	−5.3	−6.6	3.5
	4.798	4.1	6.5	5.5	−2.7
	43.179	−2.3	−2.0	−3.0	4.3
Quercitrin	0.020	5.9	6.2	7.2	−4.6
	0.177	−1.3	−1.9	−2.9	−5.6
	1.596	3.0	−4.1	−4.6	6.2
Quercetin	0.112	3.6	5.1	13.1	−7.8
	1.004	−2.9	−5.6	−4.6	2.4
	9.032	1.5	−2.2	2.0	3.5

**Table 5:** Pharmacokinetic parameters of the three constituents after oral administration of *P. capitatum* extract to rats (*n* = 6).

Parameters	Analytes				
	Free Gallic Acid	Total Gallic Acid	Free Quercitrin	Total Quercitrin	Total Quercetin
AUC_(0–t)_ (h·µg/mL)	56.87 ± 23.43	425.27 ± 154.71 *	1.12 ± 0.42	1.48 ± 0.67	12.95 ± 4.64 *
AUC_(0–∞)_ (h·µg/mL)	63.10 ± 29.24	518.99 ± 201.34 *	1.16 ± 0.43	1.58 ± 0.72	13.27 ± 4.81 *
MRT_(0–t)_ (h)	2.14 ± 0.11	4.07 ± 0.19 *	3.43 ± 0.22	3.40 ± 0.17	3.60 ± 0.21 *
MRT_(0–∞)_ (h)	2.81 ± 0.55	6.77 ± 0.89 *	3.88 ± 0.47	4.21 ± 0.54	3.86 ± 0.27 *
C_max_(µg/mL)	25.48 ± 11.92	95.29 ± 24.08 *	0.51 ± 0.19	0.59 ± 0.19	3.49 ± 1.01 *
T_max_(h)	0.50 ± 0.26	0.56 ± 0.29	0.58 ± 0.20	0.42 ± 0.09	1.00 ± 0.00

* *p* < 0.01 compared with the free group.

**Table 6:** Precursor/product ion pairs and parameters for MRM of the analytes and the I.S.

Analytes	Mode (−/+)	Transition (*m*/*z*)	Dwell (s)	Cone Voltage (V)	Collision Energy (eV)
gallic acid	−	169.0→125.0	0.05	35	15
quercitrin	+	449.0→303.0	0.05	20	10
quercetin	+	303.5→153.0	0.05	45	40
puerarin (I.S.)	+	417.0→267.0	0.05	40	30
